# Colovesical Fistula due to Sigmoid Diverticulitis

**DOI:** 10.1155/2023/8835222

**Published:** 2023-12-04

**Authors:** Christos K. Stefanou, Spiridon Gkogkos, Stefanos Flindris, Apostolis K. Paxinos, Thomas Tsiantis, Polyxeni Oikonomou, Kostas Tepelenis, Stefanos K. Stefanou

**Affiliations:** ^1^Department of Surgery, General Hospital of Filiates, Thesprotia, Greece; ^2^Department of Obstetrics and Gynecology, University Hospital of Ioannina, Ioannina, Greece; ^3^Department of Urology, General Hospital of Preveza, Preveza 48100, Greece; ^4^Department of Cardiology, General Hospital of Ioannina “G. Hatzikosta”, Ioannina 45500, Greece; ^5^Department of Surgery, General Hospital of Ioannina “G. Hatzikosta”, Ioannina, Greece; ^6^Department of Endocrine Surgery, Henry Dunant Hospital Center, Athens, Greece

## Abstract

**Introduction:**

The incidence of colonic diverticulosis has risen significantly. Diverticular disease is the most frequent cause of colovesical fistulas, which are uncommon complications of diverticulitis. Clinical signs, such as fecaluria and pneumaturia, are typically required to confirm its presence. Finding the cause of the disease so that the proper therapy can be started is the primary goal of a diagnostic workup rather than observing the fistula tract itself. *Case Presentation*. We present a 43-year-old man complaining of frequent urinary tract infections for six months. On CT abdomen and pelvis, a colovesical fistula was diagnosed. Surgery was performed, and after the division between the sigmoid colon and the bladder, a sigmoidectomy and an end-to-end colorectal anastomosis were performed. During the surgery, the fistula tract was not detected. The patient was discharged in excellent condition on day six, and the catheter was removed on day 10.

**Conclusion:**

In conclusion, as in our case, any patient with a urinary tract infection should be suspected of having this condition, especially if he has persistent symptoms that have not responded to standard medical care. Patients who present with fecaluria, pneumaturia, and other specific symptoms of a colovesicular fistula do not necessarily need a barium enema or cystography to confirm the presence of the fistula.

## 1. Introduction

Colovesical fistulas (CVFs) are characterized by an anomalous connection between the colon and the urine bladder. CVFs are uncommon and can result in significantly lower quality of life and even mortality due to urosepsis [1, 2]. Diverticulitis and cancer are the most frequent causes of the underlying pathology, with both conditions also contributing to congenital abnormalities, inflammatory bowel diseases such as Crohn's disease, and trauma [1, 3–5]. Typical presenting symptoms include pneumaturia, urinary tract infections, fecaluria, hematuria, and orchitis [4]. According to existing estimates, the prevalence of colovesical fistulas (CVFs) in patients with diverticular disease ranges from 2% to 18% [6]. The prevalence of this condition is higher in males compared to females, with a male-to-female ratio of 3 : 1. This disparity can be attributed to the presence of the uterus and broad ligaments that create a barrier between the sigmoid colon and bladder [7].

The classification of enterovesical fistula is based on the specific intestinal segment that is affected, including colovesical, rectovesical, ileovesical, and appendicovesical. According to the cited source, CVF accounts for around 70% of all fistulas, whereas ileovesical fistulas make up 15%, rectovesical fistulas account for 10%, and appendicovesical fistulas constitute 5% of cases [8].

While clinical identification of a CVF is possible, further measures such as imaging and endoscopy are sometimes required to ascertain the full extent of the fistula and its underlying etiology [5]. Surgical intervention is the customary approach for therapy.

## 2. Case Presentation

A 43-year-old male patient presented to the Emergency Department with a urinary infection. His medical history showed that in the previous six months, he had frequent urinary tract infections (urinary cultures: E. coli) and was treated with antibiotics according to the antibiograms. He had never been hospitalized for diverticulitis. No fecaluria was noticed.

According to his past surgeries, he had a gastric plication surgery due to obesity before eight months.

**Figure 1:**
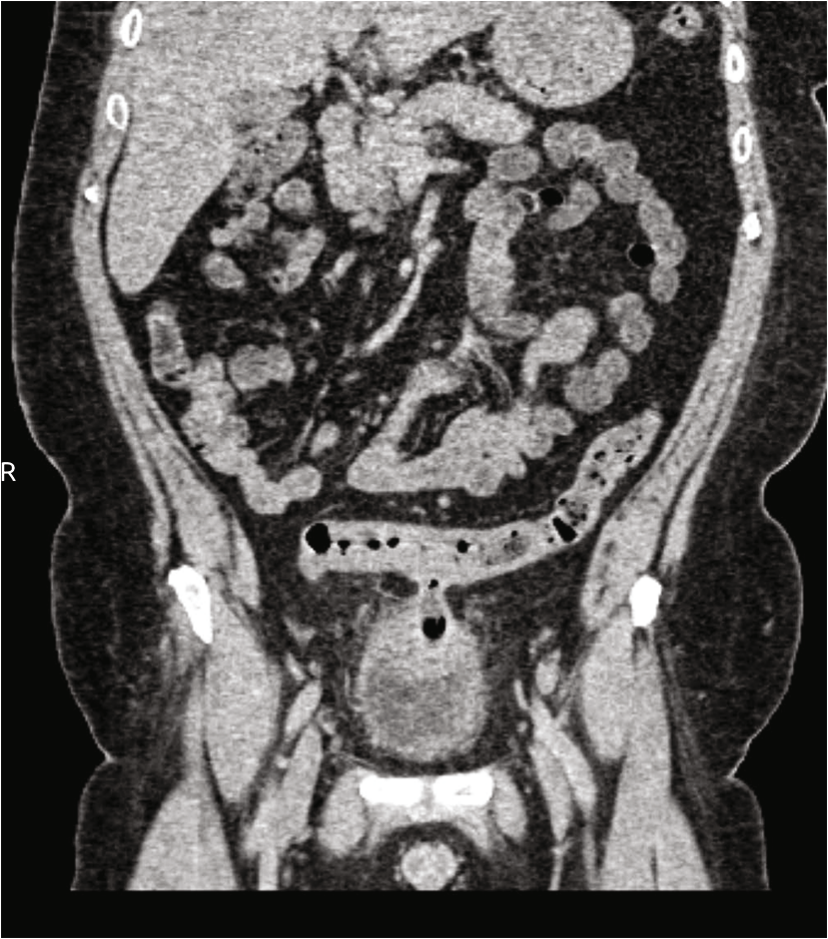
Abdominal CT. A colovesical fistula between the sigmoid colon and the bladder.

**Figure 2:**
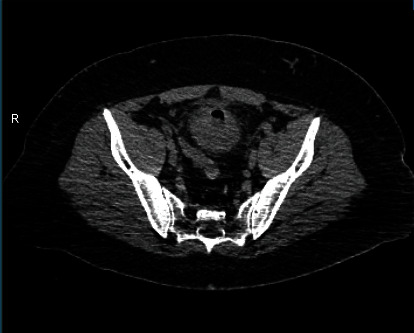
Abdominal CT. Air in the bladder.

In abdominal computed tomography, a colovesical fistula was found in contact with the dome of the bladder, and gas within the bladder was detected (Figures 1 and 2). Nevertheless, no abscess or fistula tract was observed. A cystoscopy was performed, but nothing was noticed during this procedure. Based on his medical history and the CT findings, a sigmoid colovesical fistula was suspected, and an open abdominal surgery was performed.

**Figure 3:**
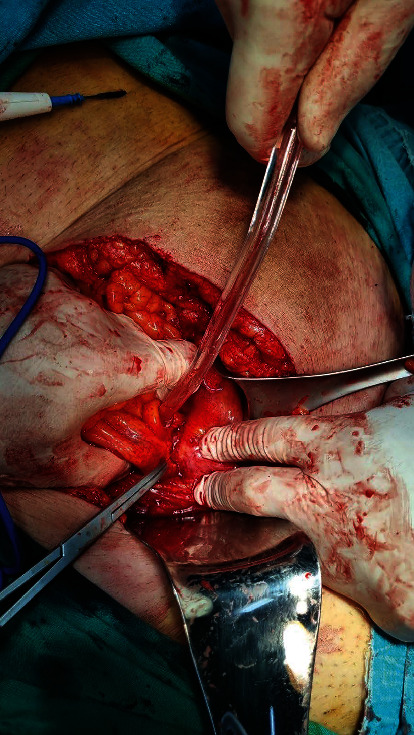
Intraoperative image. The sigmoid colon adhered to the bladder.

**Figure 4:**
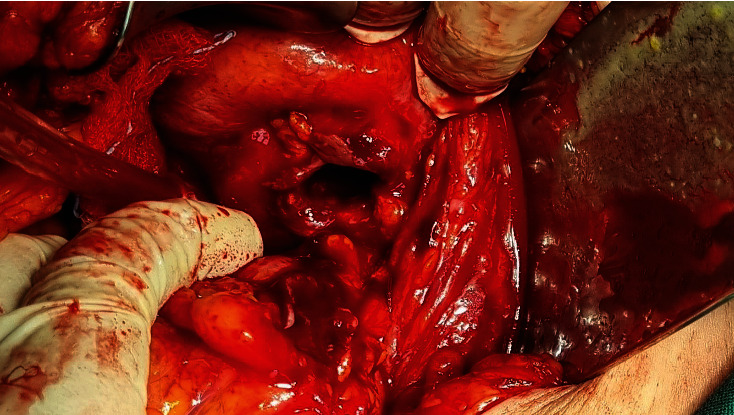
Intraoperative image. Abscess in connection with the bladder.

**Figure 5:**
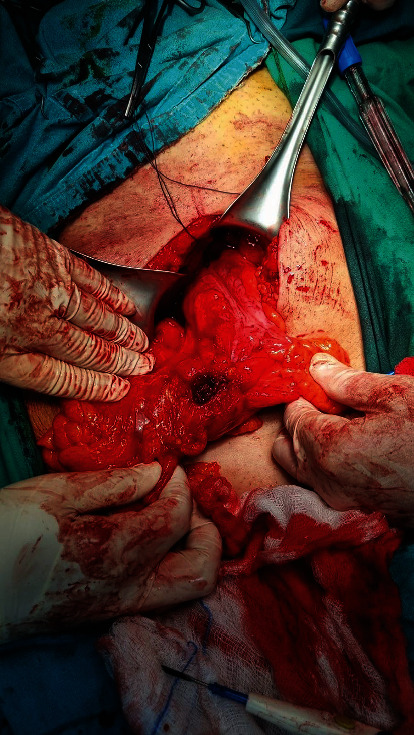
Intraoperative image. The sigmoid colon after the separation from the bladder.

In operative findings, the sigmoid colon adhered to the dome of the bladder (Figure 3). During the detachment, pus was outflow. The bladder and the sigmoid colon were separated, and an abscess was in connection with the bladder; the fistula area of the bladder was not detected (Figures 4 and 5). No leakage was observed after fluid inflow from the catheter. A sigmoidectomy was performed with an end-to-end colorectal anastomosis.

Postoperatively, no particular issues emerged. He remained in the hospital for six days and was discharged in excellent condition. The catheter was removed after ten days from the surgery.

## 3. Discussion

Colonic diverticulosis, also known as diverticulosis (presence of diverticula in the colon), is very common with advancing age and is higher in prevalence in countries with Western diets. It remains asymptomatic, while about 25% of people will develop symptomatic diverticulosis, a colonic diverticular disease [9].

Clinical presentation of colonic diverticular disease ranges from mild abdominal pain to peritonitis with sepsis. Two crucial complications of this condition are inflammation and bleeding.

Diverticulitis is defined as diverticula with inflammation and is manifested in about one-third of patients with diverticula. The pathophysiology in which a diverticulum becomes inflamed is based on obstruction of the diverticula and venous congestion, leading to perforation. The perforation's extent and localisation determine the disease's clinical behaviour [10]. When the inflamed colon erodes into adjacent organs, it causes a fistulous connection.

The most common fistulas are colovesical fistulas (the connection between the colon and bladder), and the presenting symptoms are pneumaturia and fecaluria. In a systematic review, the most common etiology of CVF is diverticulitis (87.9%); however, our patient had never previously reported about any abdominal pain [11]. The incidence of colovesical fistula in diverticular disease is approximately 2-4%. The pathognomonic finding is air within the bladder on a CT scan, which was detected in 71.4% of cases [5]. The identification of the colovesical fistula may experience a delay due to the presence of other nonspecific symptoms resulting from recurring or persistent urinary tract infections in a considerable portion of patients [5]. Fistulas can also occur, however less frequently, in relation to adjacent tissues such as the ureter, prostate, fallopian tubes, or uterus. The diagnostic methodology for colovesical fistula is of paramount importance as it serves to confirm its existence, ascertain the underlying etiology, and explore an effective therapeutic approach. The prevalence of the colovesical fistula is rather modest, with diagnostic methods including cystoscopy at a rate of 42.4%, cystography at 41.3%, barium enema at 35.3%, colonoscopy or sigmoid colonoscopy at 6.4%, and abdominal computed tomography at 30.8% [5]. When faced with cases where the existence of a fistula is uncertain, it is advisable to employ the poppy seed test as a means of confirming the condition. Subsequently, further examinations should be conducted to determine the root causes of the fistula [12]. While preoperative colonoscopy plays a crucial role in identifying the root cause, cystoscopy tends to provide nonspecific observations, such as bullous edema, which makes it challenging to evaluate the fistula tract (with a success rate of less than 50% in detecting a fistula) [13]. However, a cystoscopy is absolutely necessary to rule out urological malignancies, bladder stones, and interstitial cystitis during the investigation of CVF [13]. To assess the ureteral openings, a double J catheter may be placed before surgery in cases of severe inflammation where the risk of ureter damage is high [5].

When compared to other techniques, a CT scan has the advantage of being able to show the accompanying soft tissue masses that form the fistula between the bladder and the bowel, identify intestinal diverticula, find adhesions between the intestine and the bladder, demonstrate abscess formation, and enable tumor staging for surgical management. For some authors, the most sensitive and noninvasive examination is a CT scan, with 60-100% diagnostic accuracy [14].

Colonoscopy is important and should be performed to evaluate the colon lumen and determine the underlying etiology of fistula. Therefore, it is recommended to utilize colonoscopy or flexible sigmoidoscopy as diagnostic tools for determining the root cause of a CVF, rather than solely focusing on the identification of the fistula itself. An endoscopy is considered the most optimal method for assessing the colonic mucosa and is also recognized as the most precise approach for diagnosing colon cancer [15]. Biopsies may be conducted in cases where a potentially malignant or abnormal lesion is identified, in order to assist in the process of medical intervention and treatment.

The primary objectives of colovesical fistula surgery involve the surgical separation of the colon and bladder, followed by the subsequent procedures of sigmoidectomy and fistula therapy. The complete removal of the target organ is unnecessary when there is no presence of cancer. The distal excision of the sigmoid colon should be conducted at the point where the taenia coli diverge and extend onto the upper rectum [16]. In a manner analogous to our specific scenario, that particular region was encompassed by the resection. The management of a colovesicular fistula resulting from nonmalignant conditions is contingent upon the dimensions of the fistula. Fistulas that are below the threshold of detectability do not necessitate surgical intervention; however, those over this threshold necessitate primary sutures and the insertion of the omentum as a barrier between the bladder and the colon. Subsequently, a Foley catheter is employed for a duration of one week [17].

Typically, catheters are inserted for two weeks and then removed following a cystography examination of the leakage. Although a cystography test is not necessary, it has been observed in recent research that placing a catheter for more than a week increases problems, including urinary tract infection, urine retention, and bladder atony [18].

Treatment of CVF includes nonsurgical and surgical strategy. Patients who are considered unfit for surgery are the only ones who can receive the nonsurgical treatment. The location of the colonic lesion and the patient's comorbidities decide whether CVFs need surgery. In our case due to the absence of prior laparoscopic experience, we performed an open surgery.

The conservative treatment includes bowel rest, total parenteral nutrition, antibiotics, steroids, immunomodulatory medications, and urethral catheter drainage. A case report of a malignant CVF showed the successful treatment with octreotide [8].

Laparoscopic conservative surgery is an alternative method in patients which the sigmoidectomy could not be performed. This treatment involves a straightforward fistulectomy rather than a colon resection. The fatty tissue is positioned between the sigmoid and bladder to lower the possibility of the fistula recurrence [19]. This treatment could be useful when the fistula is not due to complicated diverticulitis; otherwise, the conservative approach could not be suitable because of the high-risk failure of the technique.

The surgical management of CVFs is contingent upon the location of the colonic lesion and the presence of comorbidities in the patient. In the context of open surgery, the conventional approach to operational care often involves the removal and reconnection of the affected part of the colon, along with the closure of the bladder. The feasibility and safety of laparoscopic treatment for CVFs have been demonstrated, particularly when performed by experienced surgeons. The rates of complications associated with this approach are equivalent to those observed in open sigmoid resection procedures. The utilization of minimally invasive procedures in the field of urological surgery has been found to decrease the likelihood of surgical site infections (SSI) as compared to the conventional approach [20]. In a retrospective cohort analysis comparing laparoscopic and converted groups, the study found that the total conversion rate to laparotomy was 34.7%. The primary reason for conversion was identified as extensive fibrosis, which was the most often observed factor leading to the need for laparotomy. A total of 26.4% of patients experienced postoperative difficulties, with 21.4% of complications observed in the laparoscopic group and 37.8% in the conversion group (*p* = 0.075). The duration of hospitalization was notably reduced in the laparoscopic group compared to the control group, with mean lengths of stay of 5.8 days and 8.1 days, respectively (*p* = 0.014). There were two instances of anastomotic leakage, both seen inside the open group as reported in reference [11]. The benefits of robotic surgery in treating CVF need additional research. Robotic surgery gives enhanced vision and articulated instruments, which may confer value in dissecting severely inflamed tissue.

The complications after elective colon resection for CVF are mortality of 1-2.3%, morbidity of 6.4% to 49% with a median of 19%, and recurrence of 2.6% to 12.5% [15].

## 4. Conclusion

Patients who present with recurrent lower urinary tract symptoms and signs, including the presence of pneumaturia and fecaluria, should be considered as potential cases of CVF. The confirmation of the diagnosis mostly relies on computed tomography, although all patients are advised to have colonoscopy and cystoscopy in order to evaluate the underlying reason. The primary approach for managing a benign CVF is surgical intervention, namely, a one-step procedure involving colonic resection, followed by a period of 7-10 days during which bladder drainage is maintained. Resection following oncologic principles is necessary for patients diagnosed with malignant fistula.

## Data Availability

Data is available in this case report.
